# YOLO-Weld: A Modified YOLOv5-Based Weld Feature Detection Network for Extreme Weld Noise

**DOI:** 10.3390/s23125640

**Published:** 2023-06-16

**Authors:** Ang Gao, Zhuoxuan Fan, Anning Li, Qiaoyue Le, Dongting Wu, Fuxin Du

**Affiliations:** 1 School of Mechanical Engineering, Shandong University, Jinan 250061, China; 202022161213@mail.sdu.edu.cn (A.G.); fanzhuoxuan@mail.sdu.edu.cn (Z.F.); 202214806@mail.sdu.edu.cn (A.L.); 202006162012@mail.sdu.edu.cn (Q.L.); 2 Key Laboratory of High-Efficiency and Clean Mechanical Manufacture, Shandong University, Ministry of Education, Jinan 250061, China; 3 Key Laboratory of Liquid-Solid Structural Evolution and Processing of Materials, Shandong University, Ministry of Education, Jinan 250061, China; wudongting@sdu.edu.cn; 4 Engineering Research Center of Intelligent Unmanned System, Ministry of Education, Jinan 250061, China

**Keywords:** intelligent robotic welding, laser visual sensor, feature extraction, structured-light vision, convolutional neural network

## Abstract

Weld feature point detection is a key technology for welding trajectory planning and tracking. Existing two-stage detection methods and conventional convolutional neural network (CNN)-based approaches encounter performance bottlenecks under extreme welding noise conditions. To better obtain accurate weld feature point locations in high-noise environments, we propose a feature point detection network, YOLO-Weld, based on an improved You Only Look Once version 5 (YOLOv5). By introducing the reparameterized convolutional neural network (RepVGG) module, the network structure is optimized, enhancing detection speed. The utilization of a normalization-based attention module (NAM) in the network enhances the network’s perception of feature points. A lightweight decoupled head, RD-Head, is designed to improve classification and regression accuracy. Furthermore, a welding noise generation method is proposed, increasing the model’s robustness in extreme noise environments. Finally, the model is tested on a custom dataset of five weld types, demonstrating better performance than two-stage detection methods and conventional CNN approaches. The proposed model can accurately detect feature points in high-noise environments while meeting real-time welding requirements. In terms of the model’s performance, the average error of detecting feature points in images is 2.100 pixels, while the average error in the world coordinate system is 0.114 mm, sufficiently meeting the accuracy needs of various practical welding tasks.

## 1. Introduction

Welding is a crucial process in modern manufacturing. Traditional manufacturing methods rely on manual welding, which is not only inefficient but also unable to guarantee welding quality. This method fails to meet the requirements of modern manufacturing for high-efficiency and high-precision welding. In comparison to traditional welding, robot welding possesses inherent advantages such as consistency in welding quality, continuous and repetitive work, and accurate tracking of welds. Against this backdrop, with the development of automation, welding robots with high stability, efficiency, and the ability to work in harsh environments are gradually replacing manual welding. However, due to the inherent nonlinearity, multivariable coupling, and uncertainty in the power system [1], simple welding robots have become increasingly unsuitable for modern production [2].

Currently, the “Teach-Playback” mode [3] is widely used in robot welding, resulting in a more favorable working environment; it increases welding efficiency to some extent and ensures the consistency of welding quality. However, the “Teach-Playback” mode still relies on the worker’s judgment for determining the welding start point and welding mode. In the welding process, thermal deformation of the welded parts can easily lead to the failure of trajectory planning using linear or arc interpolation, resulting in deviation of the welding trajectory. Therefore, many scholars have applied advanced sensor technology to welding robots to achieve real-time correction of welding trajectories [4,5,6]. Among various sensors, active optical-vision sensors are more commonly used in welding tracking due to their ability to overcome complex environmental interference using light sources compared to passive vision sensors that use natural light or arc light generated during welding. The line-structured light sensor based on active optical-vision has become increasingly popular in the automatic welding robot industry because of its noncontact nature, high robustness, high accuracy, low cost, and other advantages [7,8,9]. It is worth noting that some scholars have also explored other methods to build real-time welding automation quality assessment systems for timely detection of potential defects and problems, such as weld defect detection [10] and melt pool depth prediction [11]. These methods use detection systems in different forms and employ various technical means, but they are equally effective in helping welding robots instantly adjust welding parameters to ensure the stability and consistency of the welding process, while also improving productivity and reducing energy consumption. These methods can also be used in conjunction with the vision-based weld tracking methods mentioned above to further improve weld accuracy.

The key to achieving weld seam tracking is determining how to quickly and accurately locate the feature points of the weld seam from laser images in a noisy environment [12]. In previous studies, most scholars used traditional morphological-based image processing methods to extract and locate the feature points of the weld seam [13,14,15], which can ensure a certain accuracy in the absence of welding noise or weak welding noise, but cannot deal with complex noise conditions [16]. For a long time, researchers have had to design different morphological algorithms for different application scenarios, resulting in low robustness and low production efficiency.

The development of machine learning has changed the landscape. Neural network algorithms have demonstrated powerful capabilities in automatically extracting image features, especially in the automatic welding domain where feature extraction is required under complex noise conditions. Consequently, numerous researchers have conducted extensive studies in this area. Du et al. [17] addressed atypical weld seams in high-noise environments, initially employing binarization and region scanning for image segmentation, and then using a trained CNN model to extract candidate regions, finally extracting weld seam features through searching. Xiao et al. [18] utilized a Faster R-CNN model to obtain weld seam types and ROI regions, and then applied targeted morphological methods to detect different types of weld seams. Dong et al. [19] used a lightweight deep learning network, MobileNet-SSD, to extract ROI regions on an edge GPU device to maintain high processing speed, and used morphological methods such as region growth and centerline repair to gradually obtain strip centerlines and key point locations. However, this method suffers from the problem of low detection accuracy. Zou et al. [20] designed a two-stage detector that utilized a convolutional filter tracker and a VGG neural network for coarse and fine localization of weld seam feature points, effectively resolving the issue of drift during weld seam tracking and achieving high precision. Zhao et al. [21] constructed a semantic segmentation model based on an improved VGG Net to extract laser stripe edge features, and then acquired feature point positions using morphological methods such as the gray level centroid method, least squares method, and B-spline method, enabling the model to function well in environments with strong arc interference. Zou et al. [22], to cope with high-noise environments, proposed a welding image inpainting method based on conditional generative adversarial networks (CGAN), mapping noisy images to noiseless images and integrating them into a tracker for weld seam detection and tracking. Yang et al. [23] then used the deep encoder–decoder network framework and designed a welding image denoising method to achieve automatic laser stripe extraction from welding images. The method has a strong denoising performance under strong noise such as arc light, smoke, and spatter. Lu et al. [24] also employed a semantic segmentation strategy combined with morphology, selecting BiseNet V2 as the network architecture and using OHEM to improve segmentation performance. Compared to other segmentation methods, the arc line segmentation effect was slightly inferior, but the segmentation performance in difficult-to-segment areas was significantly improved.

Most of the aforementioned methods are based on a two-stage detection approach, where the first stage utilizes neural networks for preliminary image processing and information extraction, while the second stage typically employs morphological methods or constructs new neural networks to complete feature point detection. The former approach requires the design of different recognition and localization algorithms for various types of weld seams, increasing the design complexity. The latter approach often involves extracting similar and generic low-level features from different stages of the network models, leading to a waste of time due to redundancy. Moreover, most existing research is based on traditional CNNs, which do not achieve very good results when dealing with highly imbalanced positive and negative samples in welding process images.

In recent years, the CNN’s characterization ability has been further improved with the introduction of new neural network techniques such as attention mechanism. The YOLO series of neural networks is one of the representatives in target detection and has been widely applied in various target detection fields, including pavement crack detection [25], fruit detection [26], welding quality detection [27], and more, due to its powerful feature extraction ability, lightweight network structure, and efficient detection speed. This network series has undergone several iterations and has received continuous attention and improvement. Among these versions, YOLOv5 is one of the most mature and stable, with a stable structure, easy deployment, and ease of expansion, making it highly sought-after by researchers. Additionally, the model is continuously updated and its performance is constantly improving. Based on YOLOv5, researchers have made numerous improvements and engineering practices that have yielded significant results [28,29].

However, in the welding field, there is still a lack of research applying the YOLO [30] series model to weld seam tracking. Therefore, this study proposes a feature point detection network based on YOLOv5 [31], called YOLO-weld. It classifies weld seam feature points into 16 categories based on their dissimilarity, with the center coordinates of the target detection boxes in each category representing the weld seam feature point coordinates, transforming the weld seam feature point recognition problem into a target detection and classification problem. A welding noise generation algorithm is also proposed for data augmentation of training samples to enhance the adaptability of the model to extreme weld noise. Experiments demonstrate that YOLO-weld outperforms common CNN models in terms of detection and localization accuracy for weld seam feature points and exhibits excellent generalization capabilities, operating stably under varying intensities of welding noise.

## 2. Experiment System

**Figure 1:**
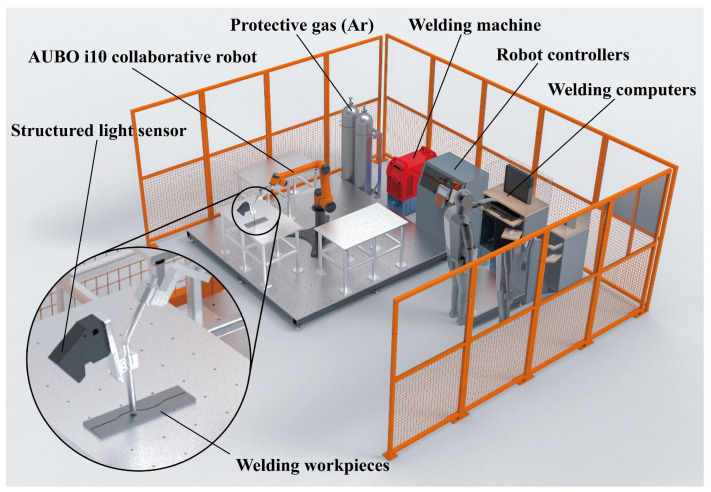
The hardware experimental platform of the optical vision intelligent welding system.

**Figure 2:**
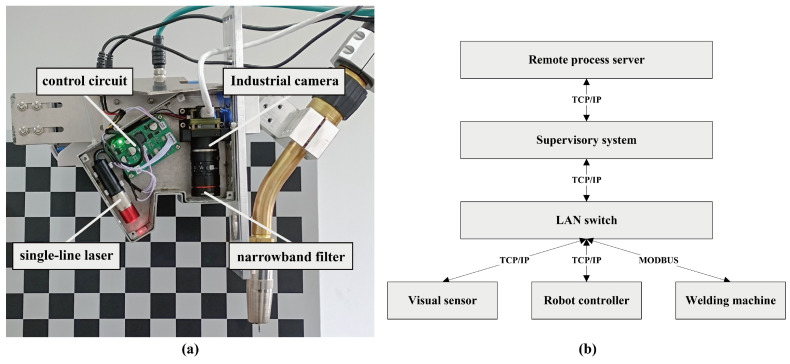
Sensor architecture and network topology diagram. (**a**) Sensor architecture. (**b**) Network topology diagram.

The hardware experimental platform of the optical vision intelligent welding system built in this study is shown in Figure 1. It is mainly composed of a robot system, a vision system, and a welding machine system. The robot system consists of the robot body, robot controller, and demonstrator. Industrial robots, while offering outstanding advantages in terms of cost and rigidity of the system, do not have anticollision mechanisms. For safety reasons, the robot used in this study is the AUBO i10 collaborative robot, which has comparable system compatibility with industrial robots and is capable of driving a 10 kg load, which meets the needs of this study. The detailed experimental hardware configuration is shown in Table 1. The visual sensor component employs a self-developed sensor, as illustrated in Figure 2a, which includes an HIK board-level industrial camera, a 660 nm narrowband filter, a laser projector emitting a 660 nm single-line laser, and a circuit for controlling the laser brightness.

The visual sensor, welding machine, and robot controller are connected to an enterprise-grade switch via a gigabit Ethernet network. The supervisory system (SS) distributes control commands for the welding process through the switch while also connecting to a remote process server via the internet to obtain process data. The network topology of the hardware system is shown in Figure 2b. In practical operation, the visual sensor acquires seam type and feature point information, transmitting it to SS. Subsequently, SS retrieves process information from the remote welding process library to direct the welding machine and welding robot in performing their tasks.

## 3. Methodology

### 3.1. Data Processing

#### 3.1.1. Setting of the Detection Targets

**Figure 3:**
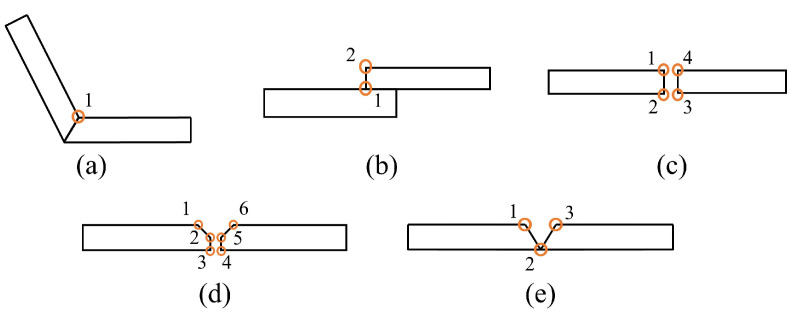
Illustration of the position of the feature points of the different weld types. (**a**) Fillet joint. (**b**) Lap joint. (**c**) Butt joint. (**d**) Y-shape. (**e**) V-shape.

To enable the welding robots to be applied in a wider range of welding scenarios, improve their ability to cope with more comprehensive and diverse weld detection tasks, and verify our model’s performance in complex and diverse welding tasks, we selected five types of welds as our detection targets: lap joint, butt joint, fillet joint, Y-shape, and V-shape. For different types of welds, different numbers of feature points were selected, and based on the dissimilarity and relative position of these feature points in the grayscale image, we further differentiated them and set each feature point as a category. Finally, all types of welds were divided into 16 feature points in total, as shown in Figure 3.

#### 3.1.2. Data Collection

**Figure 4:**
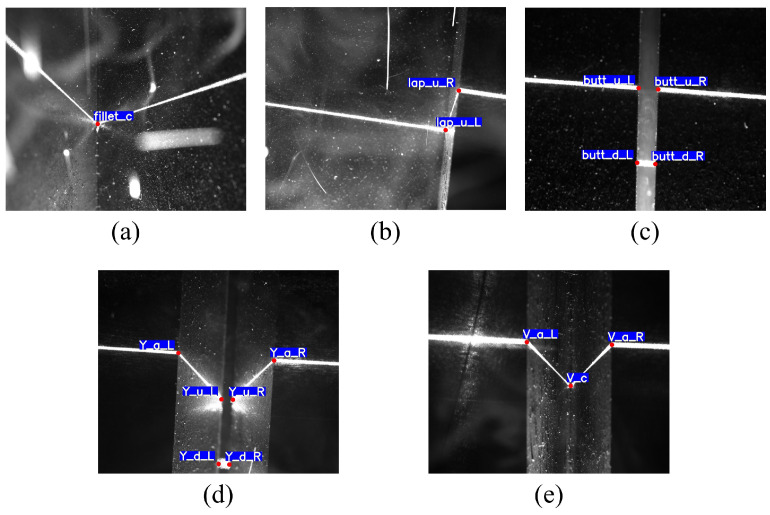
Images acquired by the sensor. (**a**) Fillet joint. (**b**) Lap joint. (**c**) Butt joint. (**d**) Y-shape. (**e**) V-shape.

First, we conducted welding on various types of weldments through manual teaching. During the welding process, we utilized our self-developed laser sensor to collect grayscale images (Figure 4). Subsequently, we performed a mean filtering operation on the original images in order to minimize the effect of imaging noise on the training results. In addition to this, since the image input accepted by the neural network is generally square, a central cropping of the original image is also performed to fit the network input. The final dataset consists of 4171 images, including 752 lap joint welds, 790 fillet joint welds, 914 butt joint welds, 761 Y-shape welds, and 954 V-shape welds. We manually labeled the dataset while randomly dividing it into a training set and a validation set in a ratio of 8:2 without a distinct test set. Finally, we manually annotated these images.

#### 3.1.3. Data Augmentation

To improve the generalization ability of the model, we applied various data augmentation techniques during the training process, including rotation, translation, scaling, vertical flipping, affine transformation, and brightness adjustment. In addition, during actual welding processes, noise such as metal spatter, smoke, and arc light often appear concentrated and continuous over time. Traditional feature point detection models have weaker recognition capabilities for strong noise images and often fail in such environments, resulting in deviations in weld trajectory.

To address the strong noise environment mentioned above, this study proposes a welding noise generation method (WNGM). This method simulates noise generated during welding processes, such as metal spatter and arc light, to enhance images and improve the model’s resistance to welding noise interference. The algorithm workflow is shown in Algorithm 1. The process of adding noise in WNGM is as follows. First, welding spatter is simulated on the input image by generating straight line segments of varying lengths, thicknesses, and densities on a new layer, followed by truncation and blurring before being added to the original image. Second, salt-and-pepper noise is added with varying degrees of aggregation to simulate smoke and dust noise. Third, to simulate image blur caused by camera focus variation, Gaussian blur is applied to the entire image. Finally, we integrate these modules to construct a welding noise generation module that can automatically adjust the random parameter generation ranges for each module with given parameters, achieving the purpose of easily controlling noise intensity.
**Algorithm 1** Welding noise generation method (WNGM)1:**procedure** WNGM(src_image)2:    process_img←zerosofthesamesizeassrc_image3:    process_img←ArcLightSpatter(process_img)4:    Add smoke noise5:    Apply Gaussian blur to the image6:    src_image←src_image+process_img7:    **return** src_image8:**end procedure**9:**function** ArcLightSpatter(image)10:    Generate random linear light streaks11:    Rotate and truncate the light streaks12:    Apply Gaussian blur to the streaks13:    **return** image14:**end function**

**Figure 5:**
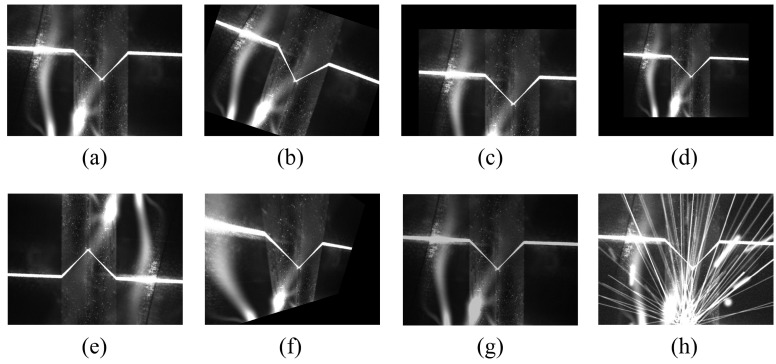
Image data augmentation. (**a**) Original image. (**b**) Rotation. (**c**) Translation. (**d**) Scaling. (**e**) Vertical flipping. (**f**) Affine transformation. (**g**) Brightness adjustment. (**h**) WNGM.

After a series of image enhancements, the final noise effects of the training images are shown in Figure 5. It can be seen that the addition of the WNGM on top of the basic data augmentation methods generates a large amount of noise, effectively simulating extreme noise conditions in the welding environment.

### 3.2. YOLOv5 Network Architecture

**Figure 6:**
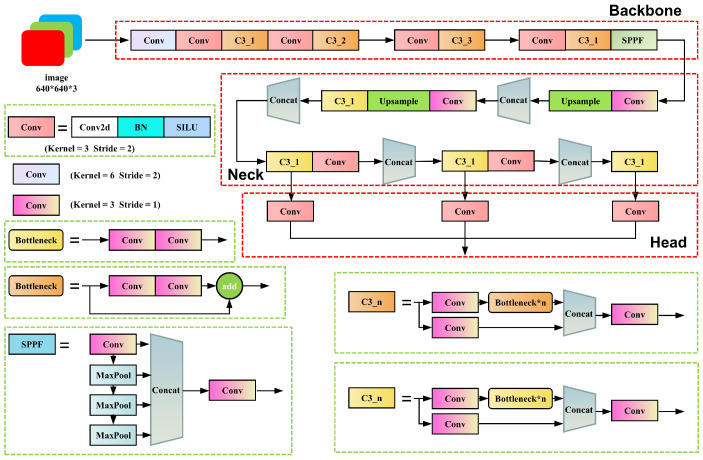
YOLOv5 network structure, with the network structure in red boxes and the specific implementation of the modules in the network in green boxes.

YOLOv5 mainly consists of three parts: backbone, neck, and head. The architecture of the YOLOv5s network is shown in Figure 6. The backbone is responsible for extracting feature information from images. The first layer of the network utilizes a convolution module with a 6 × 6 large convolution kernel to compress the width and height information of the image into channel information, reducing the number of network layers and parameters while expanding the base-level receptive field and maintaining the extraction of feature accuracy as much as possible. The C3 module is the most critical feature extraction module in this network, which essentially serves as a residual module. It divides the input information into two parts; one part passes through the bottleneck module to extract deep features, while the other part passes through only a single convolution module. Finally, the two parts are fused to complete the feature extraction. The SPPF module acquires local features at different scales through multistage max-pooling and concatenates them to increase the receptive field without changing the feature map size. The neck section adopts the PANet structure, which, through top-down and bottom-up pathways, fuses the high-level semantic features and low-level localization features extracted from the backbone network, enhancing the detection effect of different-sized objects and integrating them into different-scale heads. The head is responsible for information output. Different-scale heads have different sizes of feature maps corresponding to different-sized objects. Each grid of the feature map outputs a preselected piece of information, including the predicted category, object confidence, and bounding box center coordinates along with its width and height. Finally, the predicted objects are obtained by filtering these preselected boxes using the nonmaximum suppression (NMS) method based on their confidence and position information.

YOLOv5 has three components to the loss function: box loss, object loss, and class loss, where object loss and class loss are computed using binary cross-entropy loss, and box loss is computed using complete IoU (CIoU) loss [32].

YOLOv5 is divided into several models according to the depth and width of the network, ranging from the model with the smallest to the largest number of parameters: YOLOv5n, YOLOv5s, YOLOv5m, YOLOv5l, and YOLOv5x. We chose YOLOv5s as the base model after comparing the speed and accuracy of different models. The relevant parameters of YOLOv5s used in this study are shown in Table 2.

### 3.3. YOLO-Weld

**Figure 7:**
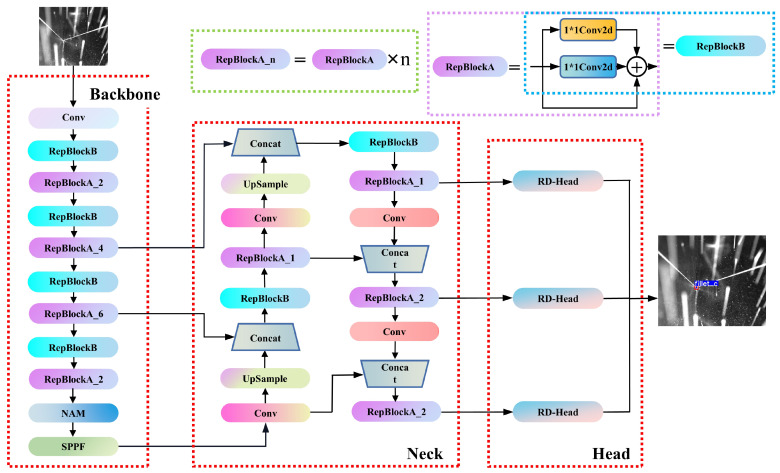
YOLO-weld network architecture.

The YOLO-weld proposed in this study is a single-stage feature point detection network based on YOLOv5, as shown in Figure 7. To better adapt to welding tasks, enhance real-time feature point detection, and improve localization accuracy, we made several targeted improvements. First, the network integrates the reparameterized VGG network, RepVGG [33], leading to the replacement of the C3 module and 3 × 3 Conv modules, originally part of YOLOv5, in the backbone network with RepVGG Block (the basic module in RepVGG). Experimental results show that this adaptation better suits GPU inference and improves inference speed with almost no loss in performance. Next, we introduced a lightweight attention module (NAM [34]) into the network, which helps highlight essential features and enhances the network model’s detection capabilities in noisy environments without introducing additional parameters. At the same time, we designed a lightweight decoupled head based on the RepVGG Block, named RD-Head, which significantly improved the model’s convergence speed and the regression effect of the predicted boxes. In the final output section, we used the center points of the predicted boxes output by the model as the weld feature points and filtered the center points using the bounding box information to obtain the final prediction results. Except for some network structure improvements, other settings, such as the loss function used by YOLO-weld remain the same as in YOLOv5s.

#### 3.3.1. RepVGG

The process of making models lightweight is a prevalent task in industrial applications. While the original YOLOv5 network uses the C3 module, which, based on CSPNet [35] design implementation and boasts powerful feature extraction capabilities, also introduces a large number of multibranch designs and model parameters, increasing the computational complexity of the network and leaving room for speed improvement. To address these issues, many researchers have replaced it with lightweight networks such as ShuffleNetV2 [36] and MobileNetV3 [37] in the backbone. Although these networks reduce floating point operations (FLOPs) to some extent, they also significantly increase the cost of GPU memory access, making it impossible to achieve speeds on the GPU that match the reduced FLOPs.

In contrast to the methods mentioned above, we introduce RepVGG, which utilizes a VGG-like architecture combined with structure reparameterization technology. During the training phase, a multibranch architecture comprising a 3 × 3 Conv, an identity branch, a 1 × 1 conv, and batch normalization (BN) layers is utilized, augmenting the network’s feature extraction capabilities while circumventing the issue of vanishing gradients. In the inference phase, structure reparameterization technology seamlessly transforms the aforementioned module into a 3 × 3 Conv. The rectified linear unit (ReLU) activation function, in conjunction with the subsequent layer, constitutes the entire inference phase of the RepVGG Block. This single-path architecture enhances parallelism and diminishes memory requirements on GPUs, resulting in accelerated inference speeds.

**Figure 8:**
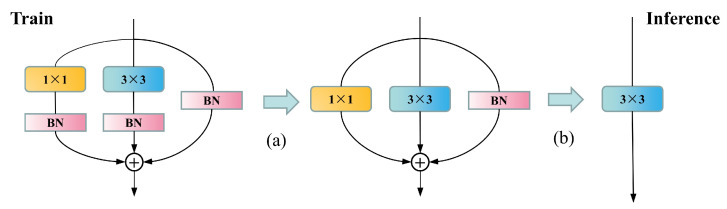
RepVGG reparameterization process, divided into two steps. Step (**a**) is the fusion of the Conv layer with the BN layers. Step (**b**) is the fusion of Conv layers from different branches.

As shown in Figure 8, the reparameterization process consists of two main steps. First, the Conv and BN layers of the same branch are fused. Their equations are expressed separately as

(1) Conv(x)=W∗x+b

(2) $$BN\left(x\right)=\left(x-\mu \right)\frac{\gamma }{\sigma }+\beta$$

Replacing the parameter *x* in BN(x) with Conv(x) yields the following equation:

(3) $$\begin{array}{cc} BN(Conv(x)) & =\left(Wx+b-\mu \right)\frac{\gamma }{\sigma }+\beta \\ \; & =\frac{\gamma }{\sigma }Wx+\frac{(b-\mu )\gamma }{\sigma }+\beta \end{array}$$

Then, the formula for the fused convolutional layer can be expressed as

(4) $$\left\{\begin{array}{c} W^{\prime }=\frac{\gamma }{\sigma }*W\\ b^{\prime }=\frac{(b-\mu )*\gamma }{\sigma }+\beta \\ BN\left(Conv\left(x\right)\right)=W^{\prime }*x+b^{\prime } \end{array}\right.$$

where μ and δ denote the cumulative mean and standard deviation in the BN layer, respectively, and γ and β are the trainable scale factor and bias. In the above equation, *W* and *b* are used to denote the convolution kernel and bias of the original convolution, and $W^{{}^{\prime }}$ and $b^{{}^{\prime }}$ denote the convolution kernel and bias of the convolution after fusing the BN layers.

**Figure 9:**
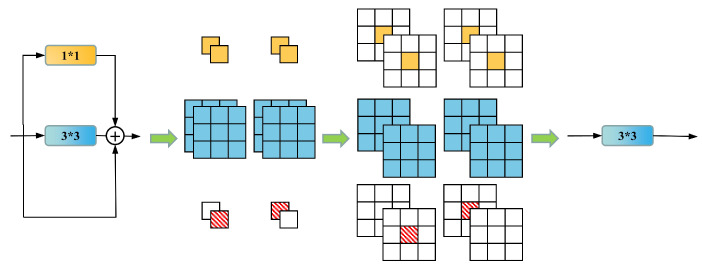
The fusion process of the different tributary Conv layers, from top to bottom, represents the transformation process of 1 × 1 Conv, 3 × 3 Conv, and identity before summation.

Another aspect is the fusion of multibranch convolutional modules, as illustrated in Figure 9. To fuse the convolutions of three branches, we first need to convert each branch’s convolution into a 3 × 3 kernel, where identity can be regarded as a 1 × 1 convolution with a unit matrix kernel, and a 1 × 1 convolution can be transformed into a 3 × 3 convolution by padding zeros around it. After completing this conversion, we add the convolution kernels and biases of the three branches together to obtain the final convolution kernel and bias.

In the network proposed by this study, RepVGG Block is divided into two categories: one is the standard feature extraction module with a stride of 1 (RepBlockA), which contains three branches during training, as shown in Figure 8. The other is the downsampling module with a stride of 2 (RepBlockB), which omits the identity branch during training and is composed of 1 × 1 and 3 × 3 convolutions.

As the RepVGG Block only comprises a 3 × 3 Conv layer followed by an ReLU layer during inference, it bears a high similarity to the downsampling module in YOLOv5’s backbone. We performed a efficient replacement of the latter with the former. Considering that the C3 module occupies the largest number of parameters in the network and has a crucial impact on the speed and accuracy of the network, we replaced the C3 module with two layers of RepVGG Block to better adapt to GPU inference, improving the inference speed while maintaining the feature extraction capability of the network model as much as possible.

#### 3.3.2. NAM

In practical welding tasks, weld images often contain a large amount of noise interference. This noise sometimes exhibits features similar to laser stripes, leading to false detections. To address this issue, we need to suppress the weights of these similar but unimportant features.

**Figure 10:**
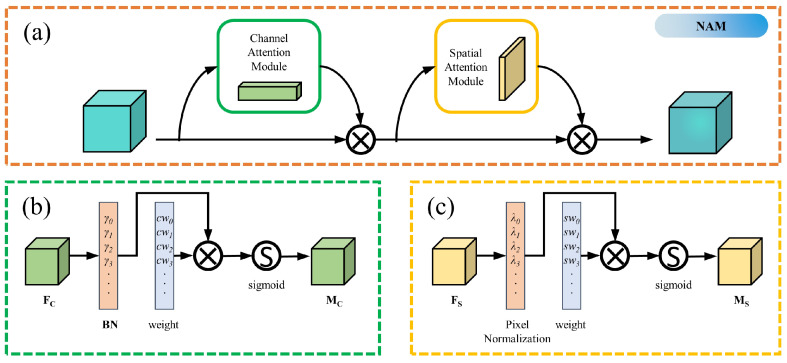
The network structure of the NAM, where, (**a**) for each input successively via the channel attention module and the hole attention module, (**b**) is the channel attention module structure and (**c**) is the spatial attention module structure.

The normalization-based attention module (NAM) used in this study is a lightweight and efficient attention module. It draws on the modular thinking of CBAM [38] and designs separate channel attention and spatial attention modules. As shown in Figure 10, unlike CBAM’s module design, NAM does not introduce additional convolutional layers. Rather, it employs batch normalization (BN) operations directly, adjusts the weights’ standard deviation using scaling factors to emphasize their significance, and uses this as a benchmark to reweight the network. This design allows the NAM module to fine-tune the weights without introducing almost any additional parameters, making it well suited for the network’s lightweight task.

The structure of the channel attention module is shown in Figure 10b, where the weights are calculated as follows:

(5) $$cw_{i}=\frac{\gamma_{i}}{\sum_{}\gamma_{i}}$$

where $cw_{i}$ denotes the weight and $\gamma_{i}$ denotes the scale factor for each channel. The spatial attention module uses a similar treatment to the channel attention module, applying BN to the spatial dimension, called pixel normalization. Its structure is shown in Figure 10c, and the corresponding weights are calculated as follows:

(6) $$sw_{i}=\frac{\lambda_{i}}{\sum_{}\lambda_{i}}$$

where $sw_{i}$ denotes the weight and $\lambda_{i}$ denotes the scale factor of the spatial dimension. For each submodule, assuming that *x* denotes the input and *W* the corresponding weight, the output *M* can be expressed as

(7) M=sigmoid(W(BN(x)))

In our network, the NAM module was added before the SPPF layer in backbone, adapting the feature extraction of the network backbone to better find feature points location.

#### 3.3.3. RD-Head

In the method proposed in this study, the weld seam type identification and feature point localization tasks are transformed into the classification and bounding box regression tasks for feature points in object detection. The prediction information is output in the head section of the network model. The original YOLOv5 head module uses a coupled head design, which simply performs a convolution operation on the input features to obtain the prediction results. This operation causes the classification and regression tasks, which are different, to use similar amounts of parameters in the prediction, leading to spatial misalignment and reduction of the model’s accuracy [39]. YOLOX [40] solves this problem by decoupling the detection head and demonstrates, through comparative experiments, that this strategy improves the network’s convergence speed and prediction accuracy. However, the decoupled head used in YOLOX introduces a large number of parameters, significantly affecting the network’s inference speed, and is not suitable for our tasks.

**Figure 11:**
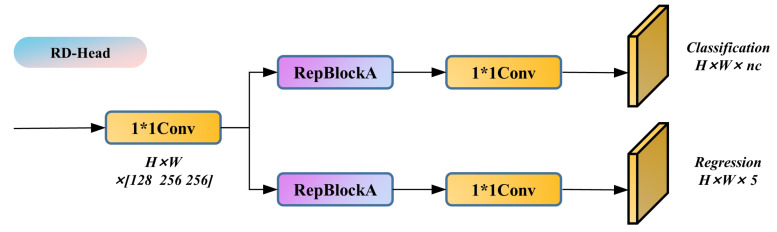
RD-Head structure diagram.

We aimed to strike a balance between the model’s inference speed and accuracy by constructing a novel decoupled head based on RepBlock, referred to as RD-Head, as illustrated in Figure 11. Given the features input from the neck, a 1 × 1 Conv is initially employed to adjust the feature channel count, and is subsequently directed to two parallel RepVGG modules with a stride of one for regression and classification tasks, respectively. Ultimately, a 1 × 1 Conv layer is applied to each branch to acquire the corresponding predictions and concatenate them, yielding an output shape identical to the original detection head. The classification branch produces categorical information, whereas the regression branch provides bounding box dimensions, center point positions, and object confidence. Subsequent experiments demonstrate that the proposed RD-Head significantly enhances network performance.

## 4. Experiment and Analysis

### 4.1. Training of the Model

The model proposed in this study was designed based on the deep learning framework Pytorch. The operating system used in this study is Ubuntu 18.04 with 43 GB RAM, the CPU is Intel(R) Xeon(R) Platinum 8255C CPU @ 2.50 GHz, and the GPU is RTX 2080 Ti. The above platform was used to train and test the models in this study and the comparison models.

In the model training, the input image was resized to 640 × 640 and data enhanced, after which it was used as the network input. We used the stochastic gradient descent (SGD) optimizer in the Pytorch framework, with the initial learning rate set to $1\times 10^{-2}$, and used a weight decay strategy to gradually decay the learning rate during training until it was reduced to $1\times 10^{-2}$ times the initial value. The default value of loss gain was chosen, the batch size was set to 8, and the number of training rounds was set to 500.

### 4.2. Training Results and Evaluation

**Figure 12:**
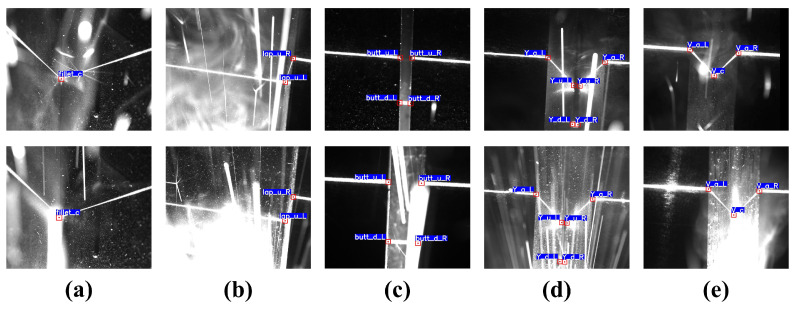
Results of the prediction of feature points of weld images using YOLO-weld. (**a**) Fillet joint. (**b**) Lap joint. (**c**) Butt joint. (**d**) Y-shape. (**e**) V-shape.

To better evaluate the performance of the model, we employed the trained model for inference on the test set. As shown in Figure 12, our model is capable of effectively extracting the class and location information of feature points for different types of weld seams under various noise conditions. Even when the feature point locations in the images are partially obscured by extreme welding noise, the model can still infer the locations of the feature points through analysis of global features, enabling the model to maintain high accuracy and robustness in strong noise environments.

To further quantify the model’s performance, we used the frames per Second (FPS) metric to assess the model’s real-time capabilities, while employing precision, recall, and mAP (mean average precision) metrics to evaluate detection accuracy [27]. Precision is used to evaluate the accuracy of the object predictions, and recall is used to assess whether all objects have been detected. Their calculation formulas are as follows:

(8) $$Precision=\frac{TP}{TP+FP}$$

(9) $$Recall=\frac{TP}{TP+FN}$$

(10) $$mAP=\frac{1}{N}\sum_{c=1}^{N}AP_{c}$$

In Equations (8) and (9), samples with annotation boxes near the predicted boxes and an IoU value greater than the set IoU threshold are considered correctly predicted samples. TP represents the number of samples that should be classified as positive and are correctly classified as positive, FP represents the number of samples that should be classified as negative but are incorrectly classified as positive, and FN represents the number of samples that should be classified as positive but are incorrectly classified as negative. In Equation (10), N denotes the number of categories, and average precision (AP) represents the area enclosed by the precision–recall (P–R) curve and represents the AP value of class *c*. The mean average precision (mAP) measures the accuracy of the model in detecting N categories, where mAP 0.5 represents the mAP value when the IoU threshold is 0.5, and mAP 0.5:0.95 represents the average mAP value when the IoU threshold increases from 0.5 to 0.95. The mAP comprehensively reflects the precision and recall of object detection. Correspondingly, in the feature point detection task studied in this study, the higher the mAP, the lower the missed and false detection rates of the graphics, which to some extent reflects higher detection accuracy.

**Figure 13:**
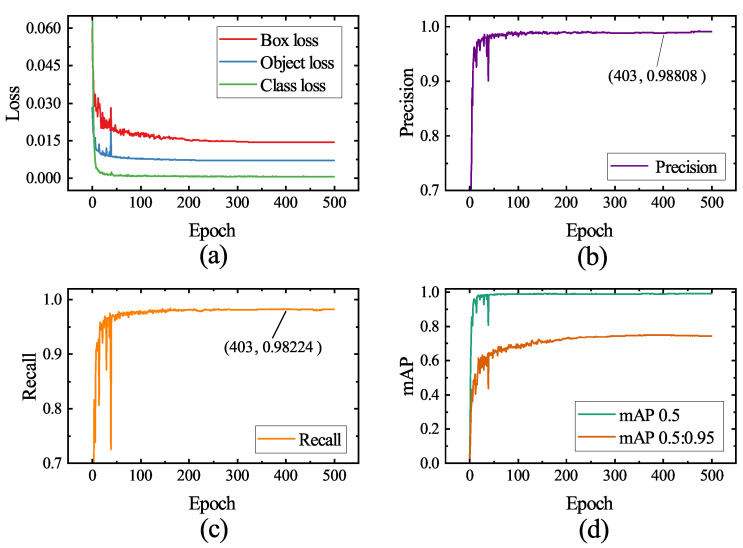
Variation curves of the validation set metrics with the number of training epochs. (**a**) Loss. (**b**) Precision. (**c**) Recall. (**d**) mAP.

The curves in Figure 13 depict the variations in loss, precision, recall, and mAP parameters on the validation set as the number of training iterations progresses. The model converges swiftly during the initial training stages, as evidenced by the rapid decrease in box loss, object loss, and class loss, as well as the rapid increase in mAP and other metrics. This occurs as the model fine-tunes the weights obtained from pretraining on a large-scale dataset, adapting to the data distribution present in welding process images. Beyond 190 epochs, the model loss approaches convergence, and the learning rate diminishes to a lower level, initiating fine-grained learning for weld seam noise images. Ultimately, at epoch 402, the model attains its peak mAP, and the weights from this epoch are chosen as the final weights for the model.

**Figure 14:**
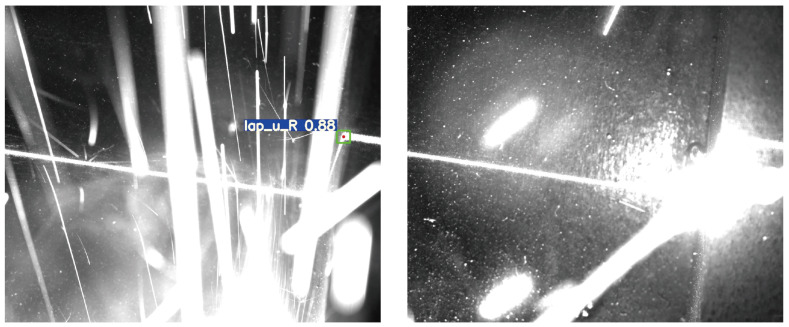
Images where feature point detection cannot be effectively performed.

Utilizing these weights to evaluate the test set, the assessment results of the aforementioned metrics are presented in Table 3. The model’s precision and recall achieve 0.990 and 0.983, respectively, signifying the proposed model’s ability to effectively extract image features and accurately detect and classify feature points. With an mAP 0.5:0.95 of 0.751, the model exhibits superior prediction outcomes under various detection standards, further emphasizing the model’s detection performance in highly noisy conditions. The difficulty to further enhance the model’s precision, recall, and mAP during training and testing arises from some images in the actual test set being affected by extreme noise, as illustrated in Figure 14. The figure reveals that intense arc light and spatter noise occupy a significant portion of the image, entirely concealing the feature points and surrounding areas, resulting in a considerably low signal-to-noise ratio (SNR) in the image. Despite the model’s robust local and global perception capabilities, it cannot make confident inferences. This observation underscores the difficulty of improving accuracy and robustness against weld feature points in the inspection process. Nevertheless, such images constitute a minimal fraction of all images; hence, the model can effectively detect weld seam feature points in highly noisy conditions. Moreover, the model’s inference speed is a mere 9.57 ms, with an FPS as high as 104.46 Hz, satisfying the real-time demands of actual industrial production processes.

**Figure 15:**
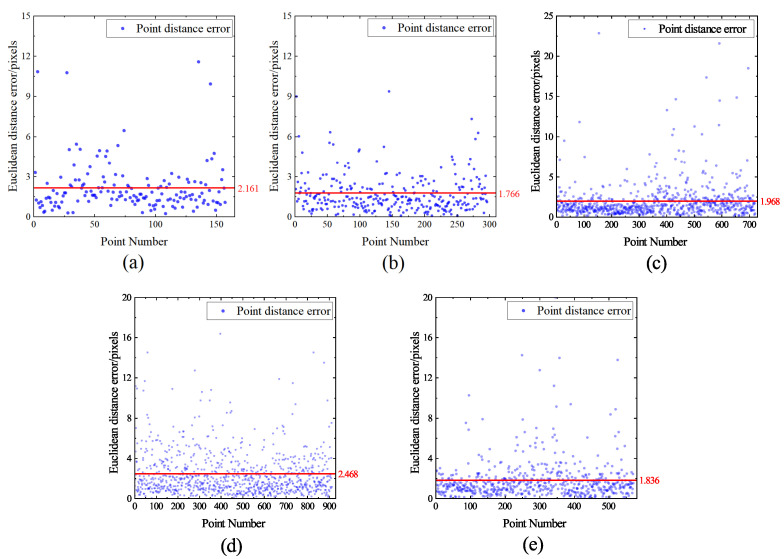
Euclidean distance distribution of predicted and marked positions for five weld types. (**a**) Fillet type. (**b**) Lap type. (**c**) Butt type. (**d**) Y type. (**e**) V type.

To provide a more intuitive reflection of the model’s accuracy in predicting feature points, we project the output coordinate information onto the original size image and calculate the deviation between the predicted and labeled coordinates on the original size image. For different types of weld seams, the Euclidean distance between the predicted and labeled positions is shown in Figure 15. Our model’s prediction deviation for most images is within 3 pixels, corresponding to an actual deviation of less than 0.15 mm. For a very small number of strong noise images (such as the bottom row in Figure 12), the feature points may be obscured, resulting in a relatively larger inference deviation for the model. Overall, the model achieves excellent performance in detecting feature points for different types of weld seams.

Moreover, to comprehensively evaluate the model’s prediction accuracy, we introduce mean absolute error (MAE) and root mean square error (RMSE) as evaluation metrics. MAE is the average absolute distance between predicted and labeled points, reflecting the deviation of predicted values from the actual values. RMSE is the square root of mean square error (MSE), which is more sensitive to outliers and better reflects the stability of the prediction system. The formulas for the two indicators are as follows:

(11) $$MAE=\frac{1}{N}\sum_{i=1}^{N}\left|\Delta e_{i}\right|$$

(12) $$RMSE=\sqrt{\frac{1}{N}\sum_{i=1}^{N}\Delta e_{i}^{2}}$$

Where *N* represents the number of samples, and $\delta e_{i}$ represents the distance difference between the predicted and standard values in different directions. When the prefixes of MAE and RMSE are X, Y, and E, they represent the deviation in the X direction, the deviation in the Y direction, and the Euclidean distance deviation, respectively. The evaluation results are shown in Table 4. It can be seen that the proposed model’s E-MAE for all predicted feature points is 2.100 pixels, and the MAE in both X and Y directions is controlled at around 1.4 pixels, indicating high prediction accuracy. The E-RMSE is 3.099 pixels.

### 4.3. Selecting the Base Model for the Experiment

YOLOv5 offers a range of models designed to accommodate tasks with varying inspection speed and accuracy requirements. In this study, we balance the real-time demands of welding and the accuracy requirements of feature point localization to select the appropriate YOLOv5 model as our base model.

We utilize various YOLOv5 models for testing on our welding dataset, maintaining consistent data enhancement and training parameters throughout the experiments. Table 5 presents the results, where “Parameter” represents the number of network model parameters, and ”Volume” indicates the memory size occupied by the model.

The comparison results indicate that despite YOLOv5n offering the highest detection speed, it is constrained by the network’s parameter count, limiting its capacity to learn features. Consequently, it exhibits comparatively low accuracy and stability in feature point localization. When the number of parameters of the network model is increased to the number of YOLOv5s, the model can learn relatively complete features and the detection accuracy is substantially improved, while the detection speed is only slightly reduced. Following this, as demonstrated by YOLOv5m and YOLOv5l, the increase in the number of parameters results in a diminished impact on the enhancement of detection accuracy and leads to a decrease in detection speed. This compromise hinders the model’s ability to satisfactorily meet the real-time demands of welding. Therefore, YOLOv5s obtains the best balance between inference speed and accuracy compared to other models with the same number of parameters. Consequently, we select YOLOv5s as our base model, upon which we improve to develop YOLO-weld.

### 4.4. Ablation Experiments

To verify the impact of the WNGM and the improved network structure on the feature point recognition and localization task, we have specifically designed two ablation experiments in this study.

**Figure 16:**
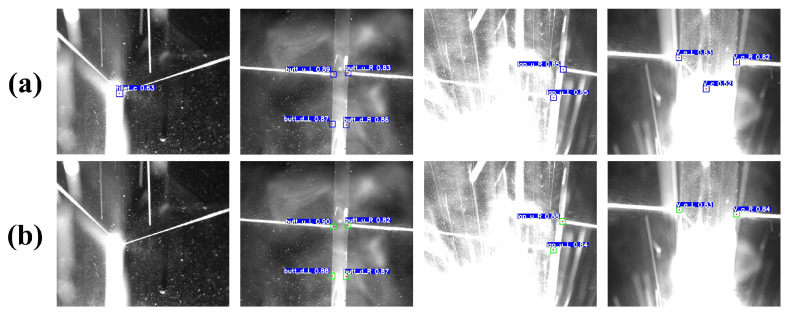
Comparative experimental images of WNGM enhancement: (**a**) with WNGM enhancement; (**b**) without WNGM enhancement.

First, we applied different data augmentation methods to the same dataset. One group did not use WNGM augmentation, while the other group used WNGM augmentation for 50% of the data. The comparison of prediction performance after using WNGM augmentation is shown in Figure 16, and the corresponding evaluation metrics are shown in Table 6. It can be observed that the model with WNGM augmentation has better robustness, and its ability to detect feature points in strong noise images is effectively improved, as well as the regression accuracy of feature points.

We subsequently conducted four experimental sets to illustrate the efficacy of the enhanced network structure. Each utilized WNGM data augmentation and maintained identical training parameters. The test results are displayed in Table 7. The improvements primarily encompass three aspects: the incorporation of the RepVGG structure, which elevates the test GPU inference speed by 56.2%, achieving 124.8 Hz, albeit with a slight reduction in feature point prediction accuracy. Subsequently, the integration of the NAM bolsters the model’s local perception capabilities and global feature extraction capacity for feature points, enabling the model to better concentrate on regions surrounding feature points. This enhances the mAP 0.5:0.95 by 0.4% while minimally impacting the inference speed. Lastly, the introduction of the RD-Head resolves the shared weights issue for classification and bounding box regression tasks in the head, effectively augmenting the prediction accuracy of bounding boxes. Following its implementation, the model’s mAP 0.5:0.95 increases by 2%, significantly improving the detection accuracy and stability of feature points. Ultimately, our proposed YOLO-weld model, in comparison to the baseline YOLOv5s model, attains a 30.8% increase in inference speed, a 1.1% enhancement in mAP 0.5:0.95, and superior feature point detection accuracy and stability.

To further validate the performance of our proposed YOLO-weld model and the generalizability of the improved method, we conducted additional experiments on VOC2007, an open-source dataset widely used for target detection.The experiments involve a comparison between YOLOv5s and YOLO-weld, using identical training parameters. The training is halted if there is no growth in the mAP values on the validation set within 100 epochs.

**Figure 17:**
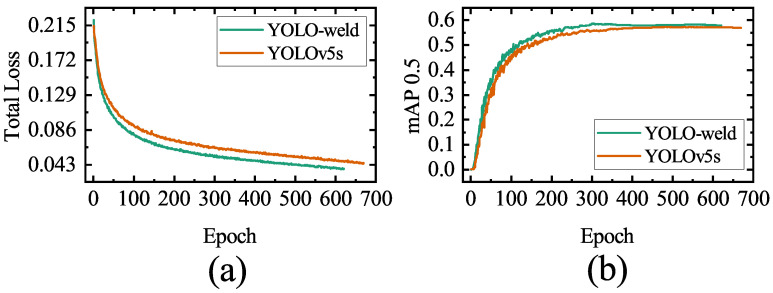
Comparison of parameter variations over epochs in the test based on the VOC2007 dataset. (**a**) Total loss. (**b**) mAP 0.5.

The variation of mAP 0.5 and total loss with the number of training rounds for both models is shown in Figure 17. Notably, YOLO-weld, an improvement over YOLOv5s, exhibits faster convergence, higher optimal accuracy, and superior regression performance. The test results of the model on the test set are shown in Table 8. The YOLO-weld proposed in this study has a slightly lower recall compared to YOLOv5s, but both precision and mAP are significantly improved, and the detection speed is significantly enhanced. Consequently, YOLO-weld also demonstrates enhanced stability and performance in nonwelding target detection tasks, further substantiating its advanced, stable, and scalable characteristics.

### 4.5. Comparative Experiments

To demonstrate the superiority of our proposed network, we compared the feature point detection model designed in this study with other neural network models. The comparative experiments used the same dataset and data augmentation methods, selected default training parameters, trained for 500 epochs, and finally performed validation and testing on the constructed validation set.

**Figure 18:**
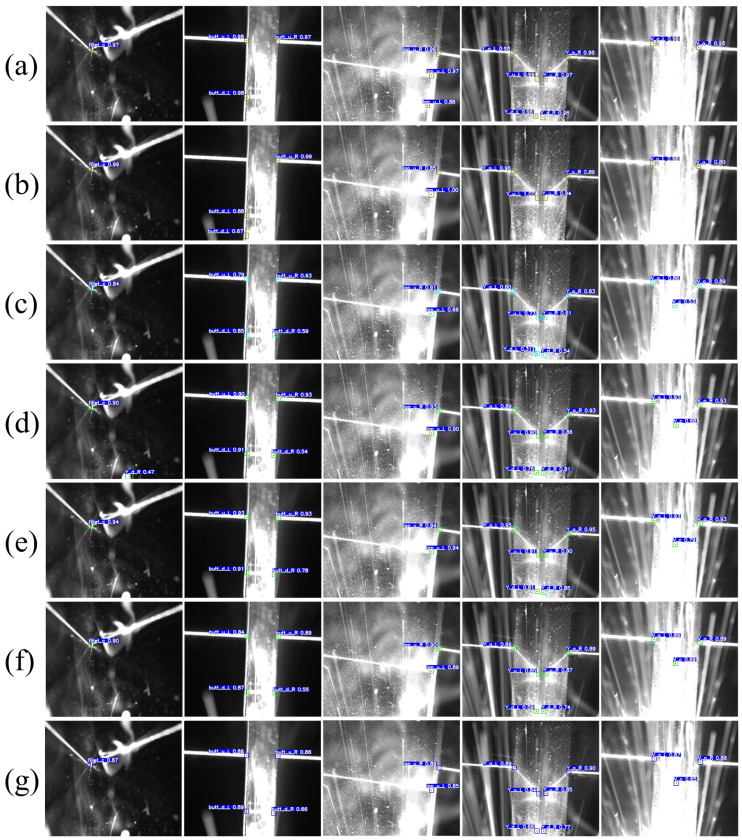
Comparison of YOLO-weld with other neural network models. (**a**) Faster RCNN. (**b**) SSD. (**c**) Center Net. (**d**) YOLOv4s. (**e**) YOLOv5s. (**f**) YOLOv7. (**g**) YOLO-weld.

The test results are shown in Figure 18, and the evaluation results are presented in Table 9. Among the compared models, Faster RCNN is a common two-stage network. The two-stage design increases the model’s training and inference costs. Simultaneously, due to the constraints of the backbone network structure, the detection accuracy is relatively poor, and there are many misidentifications. SSD adopts the prior box method from Faster RCNN and uses an end-to-end design, which improves the model’s detection speed. However, as shown in Figure 18b, the model still cannot accurately recognize feature targets in strong noise environments. CenterNet abandons the use of prior boxes and instead predicts the center point of the bounding boxes through heatmaps and regression offsets. From the actual detection data, the heatmap-based method provides the model with excellent stability, but the overly slow inference speed cannot meet the real-time requirements of welding tasks. YOLOv4 and YOLOv5, as classic models of the YOLO series, significantly improve detection speed while further enhancing prediction accuracy. YOLOv7, as the most advanced object detector currently, performs better than YOLOv5 on the COCO dataset but does not achieve a significant performance advantage in welding detection tasks and causes some loss of detection speed. In contrast, our proposed YOLO-weld model is based on the high-performance YOLOv5 and has been improved for seam feature point detection tasks. The modified YOLO-weld achieves the fastest inference speed and obtains a more significant improvement in feature point detection accuracy and stability, effectively meeting the needs of welding tasks.

### 4.6. Welding Experiment

**Figure 19:**
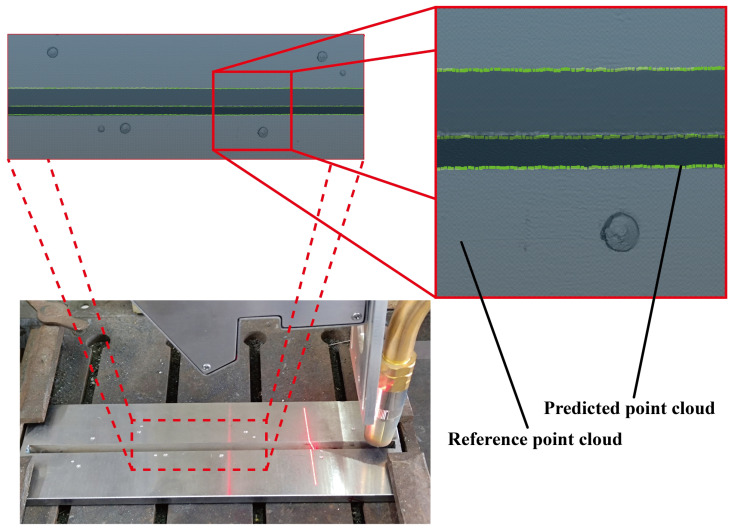
Reference and predicted point clouds collected from welding experiments.

To better evaluate the performance of YOLO-weld in practical welding tasks, V-shape welds were selected for welding experiments. First, a dense 3D point cloud was obtained as the reference by scanning the welds using the Zeiss COMET L3D 2 system, as shown by the gray portion in Figure 19. Subsequently, continuous welding images were captured during the actual welding process, and YOLO-weld was employed to perform inference on these images. Finally, the predicted coordinates were transformed into the world coordinate system, as illustrated by the green portion in Figure 19. It can be observed that YOLO-weld is capable of making accurate predictions for the feature points of the V-shape welds.

**Figure 20:**
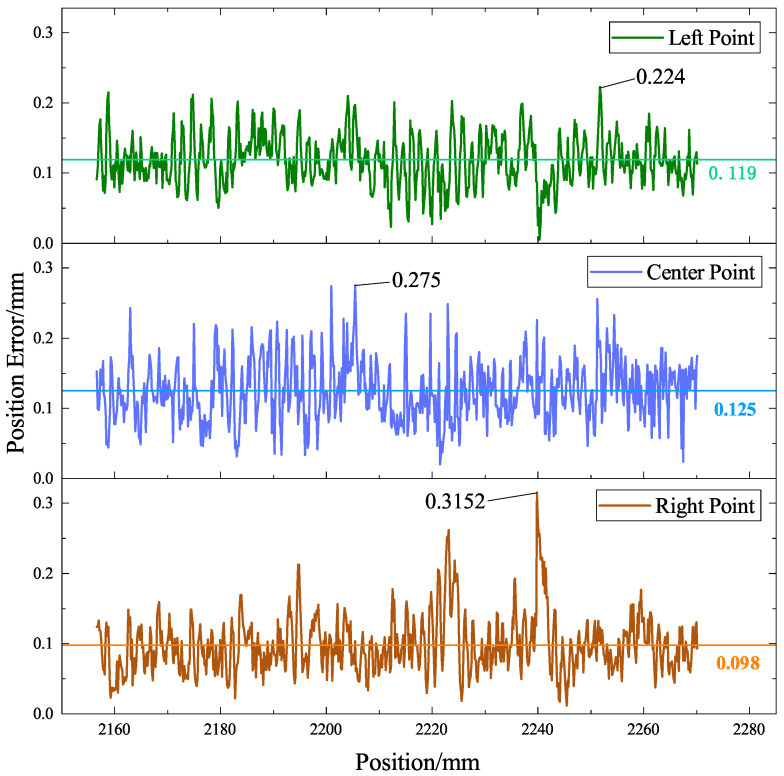
The YOLO-weld model is used to infer the error distribution of a V-shape weld in the world coordinate system.

To further quantify the performance of the model, the Euclidean distances between the point cloud of the weld feature points predicted by YOLO-weld and the reference point cloud results were calculated. The comparison results are shown in Figure 20. As can be seen from the figure, the three feature points of the V-shape welds have high accuracy. The average position error of the left feature point is 0.119 mm, with a maximum error of 0.224 mm; the average error of the center feature point is 0.125 mm, with a maximum error of 0.275 mm; and the average error of the right feature point is 0.098 mm, with a maximum error of 0.3152 mm. The average error of all feature points is 0.114 mm, which adequately meets the practical welding requirements and reflects the ability of the proposed model to overcome noise interference and ensure accurate recognition of the weld feature points.

## 5. Conclusions

This study initially examines the shortcomings of traditional two-stage weld feature point detection methods and conventional CNN techniques, such as limited inference speed and incapacity to address the severe imbalance between positive and negative samples. These constraints hinder the performance of these methods in practical weld feature detection tasks. To more effectively fulfill the real-time and accuracy demands of welding robots, we propose a single-stage, enhanced approach based on the state-of-the-art YOLOv5 model. The following conclusions were drawn:

1. A welding noise generation method was proposed for data augmentation of welding images, effectively enhancing the model’s detection capability in extreme noise environments.
2. The YOLO-weld network based on the YOLOv5 model was proposed. RepVGG was incorporated into the YOLOv5 network to improve the network detection speed while maintaining prediction accuracy. Furthermore, an efficient lightweight attention module, NAM, was introduced to enhance the network’s ability to sense feature points. In addition, the designed RD-Head employs lightweight operations to decouple the detection head, solving the spatial misalignment problem and improving detection accuracy.
3. Through experiments, the proposed YOLO-Weld model achieved a recall rate of 99.0% on a custom dataset, an mAP 0.5:0.95 of 75.1%, and an inference speed of 104.5 Hz, outperforming both two-stage detection methods and conventional CNN approaches. It is capable of accurately predicting feature points in high-noise environments while meeting real-time detection requirements. The experiments demonstrated that the mean absolute error of the feature points in the image is 2.100 pixels, and the average error of the feature point detection in the world coordinate system is 0.114 mm, proving that the proposed model possesses sufficient detection accuracy and stability to meet the requirements of practical welding tasks.

In summary, this method utilizes state-of-the-art visual perception techniques to detect feature points in welding images with complex noise backgrounds, and its accuracy surpasses traditional two-stage detection methods and conventional CNN approaches. This significantly contributes to enhancing the welding accuracy of automated welding processes and promoting the intelligent development of welding robots.

## 6. Limitations and Future Directions

Although the YOLO-weld model proposed in this study satisfies the application conditions of real-time welding well, there are still some limitations worth discussing: firstly, the article only conducts experiments for five weld types, which is not rich enough to adapt to the changing welding environment; secondly, the RD-Head designed independently in the article does not validate its performance by designing extensive independent experiments and lacks sufficient evaluation of the module; finally, the dataset used was collected in a laboratory environment, which may have fewer factors such as noise and external interference compared to scenarios in actual industrial environments, so its performance in practical applications needs to be further tested.

In future work, we will further explore the application of the model in real industrial scenarios, and try to collect data of different weld types in different environments to build a more diverse and large dataset.

## Figures and Tables

**Table 1:** Experiment equipment.

Equipment	Model	Equipment	Model
Robot	AUBO I10	Vision sensor	Self-developed
Welding machine	AOTAI MIG-500RP	Welding material	Q235
Welding feeder	AOTAI CS-501-500	Shielding gas	Ar

**Table 2:** Relevant parameters of YOLOv5s used in this study.

Parameters	Values	Parameters	Values
Input size	640 × 640	Versions	v6.1
Model depth multiple	0.33	Params	7.05 M
Layer channel multiple	0.50	FLOPs	16.1 GFLOPs

**Table 3:** Performance parameters for YOLO-weld target detection.

Precision	Recall	mAP 0.5	mAP 0.5:0.95	Time	FPS
(%)	(%)	(%)	(%)	(ms)	(Hz)
99.0	98.3	99.1	75.1	9.57	104.5

**Table 4:** YOLO-weld errors in different directions for different weld types.

Type	E-MAE	X-MAE	Y-MAE	E-RMSE	X-RMSE	Y-RMSE
	(pixel)	(pixel)	(pixel)	(pixel)	(pixel)	(pixel)
All	2.100	1.437	1.207	3.099	2.342	2.029
Fillet	2.161	1.364	1.371	2.831	2.245	1.725
Lap	1.766	1.273	0.972	2.492	2.086	1.363
Butt	1.968	1.301	1.153	3.155	2.274	2.187
Y	2.468	1.752	1.363	3.484	2.724	2.172
V	1.836	1.211	1.101	2.700	1.869	1.949

**Table 5:** Comparative test of YOLOv5 model with different parameter sizes.

Method	Parameter	mAP 0.5:0.95	E-MAE	E-RMSE	FPS
	(%)	(pixel)	(pixel)	(Hz)	
YOLOv5n	1.78	72.1	2.325	4.181	93.3
YOLOv5s	7.05	74.0	2.202	3.586	79.9
YOLOv5m	21.9	74.3	2.115	3.527	67.8
YOLOv5l	46.2	75.0	2.075	3.365	56.6

**Table 6:** Results of comparison experiments with and without WNGM enhancement.

Method	With WNGM	mAP 0.5:0.95	E-MAE	E-RMSE
		(%)	(pixel)	(pixel)
YOLO-weld	Yes	75.1	2.100	3.099
YOLO-weld	No	74.1	2.103	3.318

**Table 7:** Results of ablation experiments with a modified YOLO-weld structure.

Method	mAP 0.5:0.95	E-MAE	E-RMSE	FPS
	(%)	(pixel)	(pixel)	(Hz)
YOLOv5s	74.0	2.202	3.586	79.9
+RepVGG	72.7	2.212	3.671	124.8
+RepVGG+NAM	73.1	2.187	3.384	121.9
+RepVGG+NAM+RD-Head	75.1	2.100	3.099	104.5

**Table 8:** Results of comparative test of model parameters based on VOC2007 dataset.

Method	Precision	Recall	mAP 0.5	mAP 0.5:0.95	FPS
	(%)	(%)	(%)	(%)	(Hz)
YOLOv5s	64.3	55.5	57.3	32.1	87.4
YOLO-weld	67.7	54.8	58.9	35.6	112.6

**Table 9:** Comparison of YOLO-weld with other network models.

Method	mAP 0.5:0.95	E-MAE	E-RMSE	FPS
	(%)	(pixel)	(pixel)	(Hz)
Faster RCNN [41]	-	6.489	7.688	26.6
SSD [42]	57.1	3.949	4.871	55.8
CenterNet [43]	71.7	2.426	3.846	20.0
YOLOv4s [44]	72.9	2.251	4.233	76.8
YOLOv5s	74.0	2.202	3.586	79.9
YOLOv7 [45]	74.4	2.131	3.672	67.2
YOLO-weld (ours)	75.1	2.100	3.099	104.5

## Data Availability

Not applicable.
